# Artificial intelligence-assisted management of retinal detachment from ultra-widefield fundus images based on weakly-supervised approach

**DOI:** 10.3389/fmed.2024.1326004

**Published:** 2024-02-06

**Authors:** Huimin Li, Jing Cao, Kun You, Yuehua Zhang, Juan Ye

**Affiliations:** ^1^Eye Center, The Second Affiliated Hospital, School of Medicine, Zhejiang University, Hangzhou, Zhejiang, China; ^2^Zhejiang Feitu Medical Imaging Co., Ltd, Hangzhou, Zhejiang, China

**Keywords:** weakly supervised, deep learning, localization, retinal detachment, ultra-widefield fundus images

## Abstract

**Background:**

Retinal detachment (RD) is a common sight-threatening condition in the emergency department. Early postural intervention based on detachment regions can improve visual prognosis.

**Methods:**

We developed a weakly supervised model with 24,208 ultra-widefield fundus images to localize and coarsely outline the anatomical RD regions. The customized preoperative postural guidance was generated for patients accordingly. The localization performance was then compared with the baseline model and an ophthalmologist according to the reference standard established by the retina experts.

**Results:**

In the 48-partition lesion detection, our proposed model reached an 86.42% (95% confidence interval (CI): 85.81–87.01%) precision and an 83.27% (95%CI: 82.62–83.90%) recall with an average precision (PA) of 0.9132. In contrast, the baseline model achieved a 92.67% (95%CI: 92.11–93.19%) precision and limited recall of 68.07% (95%CI: 67.25–68.88%). Our holistic lesion localization performance was comparable to the ophthalmologist’s 89.16% (95%CI: 88.75–89.55%) precision and 83.38% (95%CI: 82.91–83.84%) recall. As to the performance of four-zone anatomical localization, compared with the ground truth, the un-weighted Cohen’s κ coefficients were 0.710(95%CI: 0.659–0.761) and 0.753(95%CI: 0.702–0.804) for the weakly-supervised model and the general ophthalmologist, respectively.

**Conclusion:**

The proposed weakly-supervised deep learning model showed outstanding performance comparable to that of the general ophthalmologist in localizing and outlining the RD regions. Hopefully, it would greatly facilitate managing RD patients, especially for medical referral and patient education.

## 1 Introduction

Retinal detachment (RD) is a sight-threatening condition that occurs when the neurosensory retina is separated from the retinal pigment epithelium (1). Several population-based epidemiological studies of RD find an annual incidence of around 1 in 10,000 (2). It has been estimated that the lifetime risk of RD is about 0.1% (3, 4). However, early intervention facilitates the prevention of disease progression and improves prognosis. Clinically, scleral buckle, vitrectomy, and pneumatic retinopexy are the most common surgical approaches to repairing RD (5–7). Before the surgery, patients should be instructed to lie in the appropriate position to minimize the detachment extending and improve visual outcomes (6, 8, 9). Postural guidance is consistent with the localization of the lesion throughout the management. However, corresponding patient education is not often adequate in busy clinical situations which may lead to poor patient compliance (10). Therefore, an efficient and reliable method for localizing and estimating the detached retinal regions is fundamental for detailed postural instruction and medical referrals, especially in remote areas with insufficient fundus specialists.

In recent years, artificial intelligence (AI) models for RD detection based on color fundus photography (CFP) and optical coherence tomography (OCT) have been gradually established (11–14). However, the emergence of the ultra-widefield fundus (UWF) imaging system promotes the intelligent diagnosis of fundus diseases to a new height. A panoramic image of the retina with 200° views allows for detailed rendering of the peripheral retina, which compensates for the deficiency of traditional fundus images (15). Ohsugi et al. (16) made a pioneering attempt to diagnose rhegmatogenous RD with a small sample of UWF images based on deep learning algorithms. Later, Li et al. (17) proposed a cascaded deep learning system using UWF images for various RD detection and macula status discerning. Despite promising advancements, their work mainly focused on the presence or absence of the target disease. However, the concrete localization of the RD lesions, a crucial need for therapeutic decision-making including the preoperative posture and surgical options, is not fully emphasized (18–21).

Generally, the extent of the retinal lesion is obtained using the supervised models which requires elaborate labeling for most existing algorithms. Whereas, the equivocal boundaries of lesion, as well as the lack of expert annotations considerably hinder the efficient development of related models. In this context, weakly supervised learning, where the learning model can be trained with incomplete and simplified annotations, has attracted great attention (22). It typically fits for training lesion localization and segmentation models in medical images. For instance, Ma et al. (23) resorted to classification-based Class Activation Maps (CAMs) to segment geographic atrophy in retinal OCT images. Monaro et al. (24) proposed an architectural setting that enabled the weakly-supervised coarse segmentation of age-related macular degeneration lesions in color fundus images. The incorporation of lesion-specific activation maps provides more meaningful information for diagnosis with great explainability. In medical imaging, Gradient-weighted CAM (Grad-CAM) (25) is one of the most commonly used techniques to generate coarse localization maps. However, most approaches derived from it only focus on the discriminative image regions but ignore much detailed information. To alleviate this issue, Qin et al. (26) proposed an activation modulation and recalibration (AMR) scheme. The combination architecture of a compensation branch and spotlight branch could achieve better performance on image-level weakly supervised segmentation tasks. Given our purpose of achieving lesion-specific holistic localization, working under coarse image-level annotation instead of bounding box annotation is highly desirable (22, 27–29). Moreover, incorporating the AMR scheme mentioned above with our approaches could generate high-quality activation maps to compensate for previous detail-loss issues.

Therefore, we proposed a weakly supervised learning model to generate localization maps that outline the RD lesions based on UWF images. Relying on the localization maps, the potential diagnostic evidence will be instantaneously transmitted to the clinicians for reference. Furthermore, individual postural guidance will be generated for healthcare reference to the patients.

## 2 Materials and methods

This study was conducted adhering to the tenets of the Declaration of Helsinki. It was approved by the Medical Ethics Committee of the Second Affiliated Hospital of Zhejiang University, School of Medicine.

### 2.1 Data acquisition

A total of 30,446 UWF images were retrospectively obtained from visitors presenting for ophthalmic examinations between 1 May 2016 and 15 August 2022, at Eye Center, The Second Affiliated Hospital, School of Medicine, Zhejiang University. Images insufficient for interpretation were excluded, including (1) Poor-view images, referring to images with significant deficiencies in focus or illumination, visibility of the optic disc, or over one-third of the field obscured by the eyelashes or eyelids. (2) Poor-position images, referring to images with significantly off-center optic disc and macula due to incorrect gazing in the image capture process. The UWF images were captured using an OPTOS nonmydriatic camera (OPTOS Daytona, Dunfermline, United Kingdom) with 200-degree fields of view. The subjects underwent the examinations without mydriasis. All of the UWF images were anonymized before being involved in this study.

### 2.2 Image labeling and the definition of RD regions

A professional image labeling team was recruited to generate the ground truth. The team consisted of two retinal specialists with more than 5 years of clinical experience and one senior specialized ophthalmologist with over 20 years of clinical experience.

**Figure 1:**
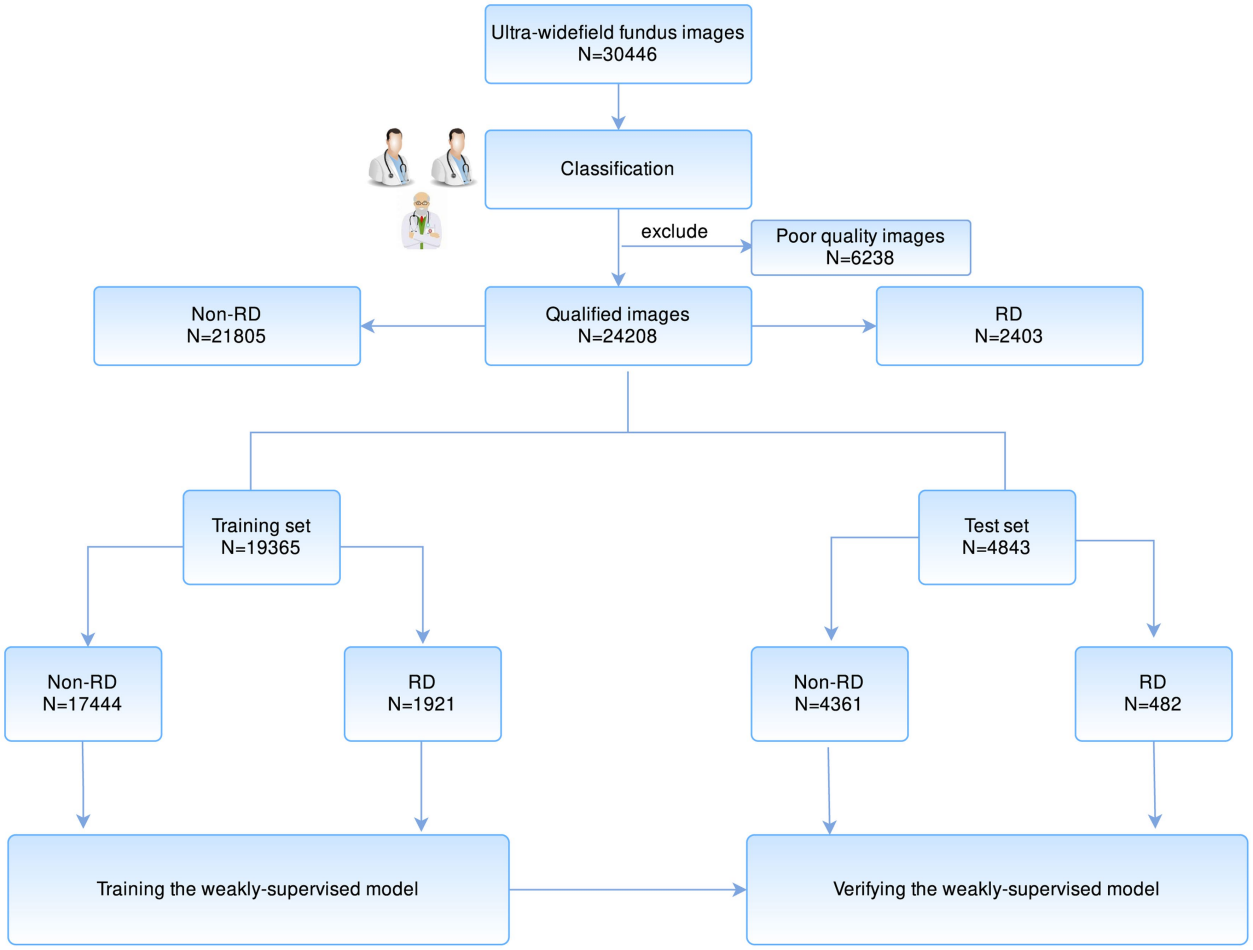
The workflow of developing a weakly supervised learning model for the localization of RD region based on UWF images. RD, retinal detachment; UWF, ultra-widefield fundus.

At first, the included UWF images were annotated with image-level labels after quality filtration. Two specialists, respectively, classified all images into two types: RD and Non-RD. The ground truth was determined based on their consensus. Any divergences were finally arbitrated by the senior specialized ophthalmologist. Figure 1 illustrates the workflow of image classification.

Then, the uninvolved fovea of each RD image (Macula ON) was marked manually to further obtain the specific anatomical zone for postural guidance. Besides, the RD regions of images in the test set were independently contoured by two specialists. The ground truth of the RD region was determined based on the intersection of their labeled areas. Any image with less than 0.9 intersection-over-union (IOU) of the labeled RD regions was confirmed by the senior specialized ophthalmologist.

### 2.3 Development of the weakly-supervised deep learning model to localize the RD regions

UWF images incorporate a vast of critical information about the profile and distribution of the lesions, which is essential for the healthcare of RD patients. Clinically, typical RD is recognized by an elevated and corrugated retinal appearance accompanied by retinal breaks, and such features can often be recognized by the deep learning algorithm. Based on this rationale, we propose a model that enables the localization of RD regions based on weakly supervised training. The design of the model consisted of two sections: localizing the RD lesions and generating postural guidance according to the anatomical zone of the lesion.

In the localization section, an attention modulation module (AMM) (26) was involved in our scheme to realize recalibration supervision and generate lesion-specific activation maps. In the first place, it was necessary to extract the fundus’ region of interest (ROI). The four corners (left and right top, left and right bottom) in a UWF image were called irrelevant areas since there was no fundus information in these four regions. These irrelevant regions from different images were variable in texture but highly similar in extent. We manually crafted an ROI template to erase pixels in these irrelevant regions. Local contrast enhancement (CLAHE) was applied to image augmentation afterward.

**Figure 2:**
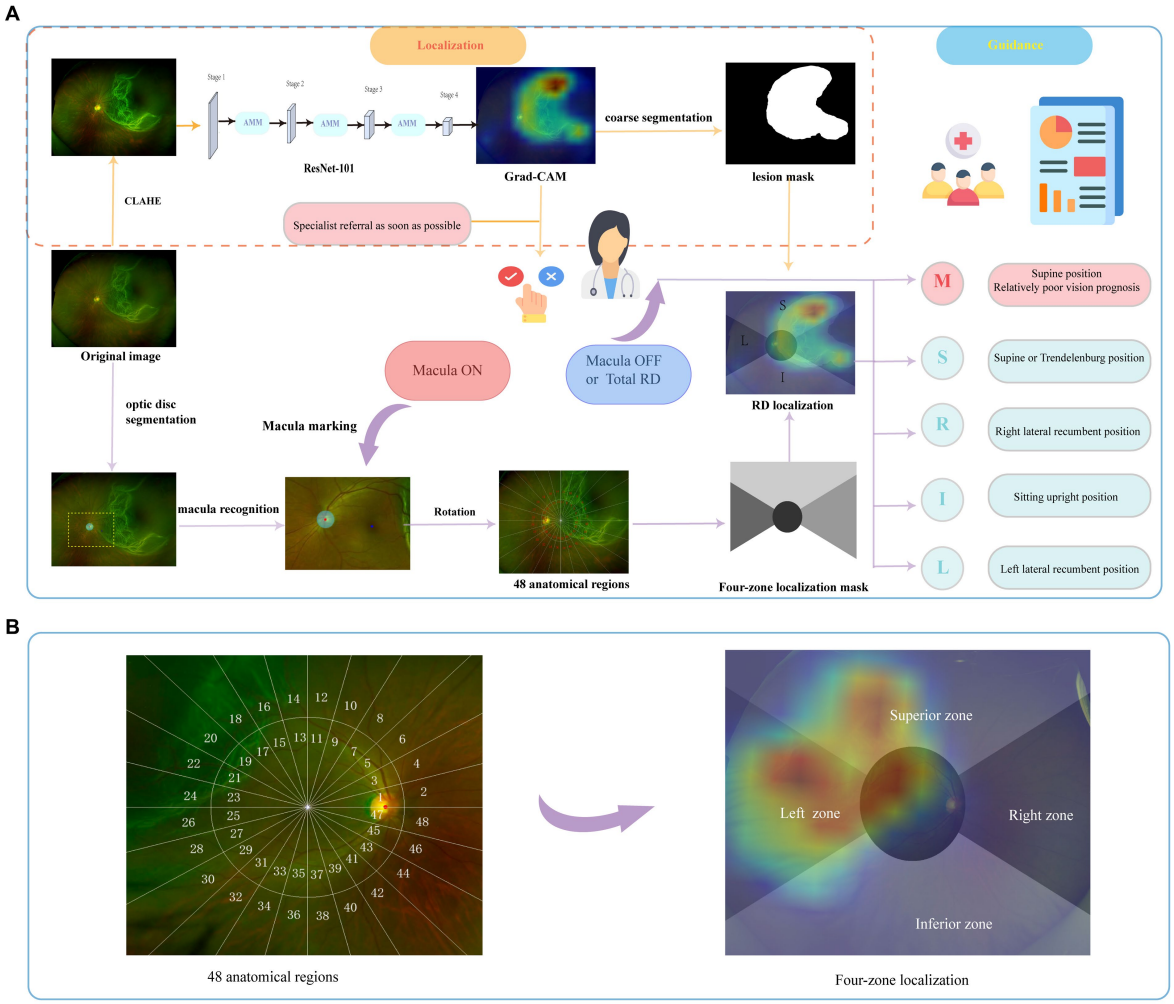
The schema of the overall study. The brief illustration of RD region localization and corresponding postural guidance **(A)**. The retina was divided into 48 anatomical regions to evaluate the holistic localization performance. The final four-zone overlaid image was generated for postural guidance **(B)**. RD, retinal detachment; M, macula zone; S, superior zone; R, right zone; I, inferior zone; L, left zone.

A ResNet-101 (30) was pre-trained to identify RD cases with a learning rate of 0.01 and focal loss (alpha was set to 0.65, gamma was set to 1.15). Then, AMM was employed to emphasize region-essential features for the segmentation task between every two stages, as shown in Figure 2. Features from the discriminative regions were considered to be the most sensitive features, and the minor features referred to features that are important but easily ignored (31). The AMM can rearrange the distribution of the feature importance to highlight sensitive and minor activations, which is crucial to generating semantic segmentation masks. The ResNet-101 with AMMs was fine-tuned with a learning rate of 0.001. Probability maps were generated based on feature maps from stage 4 by Grad-CAM and resampled to the original size afterward.

In the guidance section, the coarse segmentation of the RD region with pseudo labels obtained from localization maps with a probability threshold of 0.5 was carried out. As shown in Figure 2, a coordinate system was constructed based on the recognition of fovea marked manually and optical disc segmented by U-Net (32). Details on the established zoning principles are presented below. The primary zone label is assigned corresponding to the largest number of RD pixels. Then, the predicted label was output based on the coarse lesion segmentation results to generate the customized postural guidance.

### 2.4 Zoning principles

The panoramic retinal view is divided into four zones centered on the manually labeled macula fovea. It is calibrated with a horizontal line through the fovea and optic disc center. In a clockwise direction, we designate 2–4 o’clock as the right zone, 10–2 o’clock as the superior zone, 8–10 o’clock as the left zone, and 4–8 o’clock as the inferior zone. In addition, we pay more attention to the posterior pole, designated as the circle centered on the macula and including the optic disc (33), which is closely associated with the surgical option and visual prognosis (4, 6). To further evaluate the holistic localization performance, each zone was divided clockwise by 15° to obtain 48 anatomical regions defining the entire retina as shown in Figure 2. Each image has a 48-length vector label for 48-partition localization. The label is assigned as 1 when more than 50 RD pixels fall into this partition.

### 2.5 Sensitivity analysis

Given that difference in image resolution of input data may have impacts on the localization outcome. We implemented sensitivity analyses based on three common image resolutions including 256 × 256, 512 × 512, and 1,024 × 1,024 pixels. We evaluated the 48-partition localization performance of our weakly-supervised model in these contexts separately and selected the optimal resolution model for further evaluation.

### 2.6 Comparisons of the proposed model with the baseline model and general ophthalmologist

A comparison experiment with the proposed model was conducted using a baseline model without AMM to explore the performance enhancement that comes with the AMR scheme. Meanwhile, to evaluate our weakly-supervised deep learning model in the localization of the RD region, we recruited a general ophthalmologist with 3 years of clinical experience. It is challenging to clearly define the contours of the RD region, considering its equivocal borders, even for clinicians. Given that the final localization is the essential factor for postural instruction, we evaluated their performance of lesion body localization rather than the edge segmentation performance. According to the defined ground truth, we compared the localization performance of the proposed model with that of the baseline model and general ophthalmologist based on the test set, respectively.

### 2.7 Statistical analysis

The precision, recall, F1 score, sensitivity, specificity, and accuracy of the models and general ophthalmologist were calculated according to the reference standard. The F1 score is the harmonic mean of precision and recall, which is calculated as:

$$F1\;\mathrm{score}=\frac{2*\mathrm{Precision}*\mathrm{Recall}}{\mathrm{Precision}+\mathrm{Recall}}$$

The precision-recall curve was generated to visualize the localization performance of the deep learning models. The Cohen’s Kappa value of the model and general ophthalmologist compared with the reference standard for the four-zone localization was calculated to evaluate the consistency. All statistical analyses for the study were conducted using SPSS 26.0 (Chicago, IL, United States) and Python 3.7 (Wilmington, DE, United States).

## 3 Results

### 3.1 Data characteristics

In total, 30,446 images were obtained for preliminary model development. After filtering 6,238 poor-quality images that are insufficient for interpretation, 24,208 eligible images were annotated. Two thousand four hundred and three were classified as RD, while the remaining 21,805 images were classified as non-RD. The dataset was randomly split in 80:20 ratios according to the Pareto principle, with 19,365 (80%) images as a training set and 4,843 (20%) as a test set. The baseline characteristics of collected images are summarized in Table 1.

**Table 1:** Baseline characteristics of the training and test datasets.

	Training set (80%)		Test set (20%)	
	(*n* = 19,365)		(*n* = 4,843)	
	RD	Non-RD	RD	Non-RD
Total no. of images	1,921	17,444	482	4,361
No. of OD images	1,019	9,163	256	2,325
No. of OS images	902	8,281	226	2,036

RD, retinal detachment; OD, right eye; OS, left eye.

### 3.2 Evaluation of the weakly-supervised deep learning model to localize the RD regions

In the test set, the associated lesions of 480 RD images are successfully localized with activation maps. Only two cases have been missed due to the inconspicuous shallow detachment. In 467 Macula-ON RD images, the entire retina is divided into 48 anatomical regions based on the location of the optic disc and macula fovea, as illustrated in Figure 2, to evaluate the holistic localization of the RD region in the test set. The following anatomical localization evaluation will be specific to these 467 RD images.

Table 2 exhibited the holistic localization performance of our weakly supervised model with three image resolutions for sensitivity analysis. The results showed that the image resolution of 1,024 × 1,024 pixels had the highest precision of 89.14% (95%CI: 88.52–89.73%). However, the image resolution of 512 × 512 pixels achieved the highest recall of 83.38% (95%CI: 82.91–83.84%) and acceptable precision with an optimal F1 score of 84.81% (95%CI: 84.26–85.35%). As a result, the following localization evaluation adopted the image resolution of 512 × 512 uniformly.

**Table 2:** The holistic localization performance of our weakly-supervised model with different image resolutions.

Resolutions (pixels)	Precision (95%CI)^1^	Recall (95%CI)^1^	F1 score (95%CI)^1^
256 × 256	0.8718 (0.8653–0.8780)	0.7381 (0.7304–0.7457)	0.7994 (0.7931–0.8055)
512 × 512	0.8642 (0.8581–0.8701)	**0.8327 (0.8262–0.8390)**	**0.8481 (0.8426–0.8535)**
1,024 × 1,024	**0.8914 (0.8852–0.8973)**	0.7284 (0.7206–0.7361)	0.8017 (0.7955–0.8078)

^1^The localization performance of the weakly-supervised deep learning model was evaluated with a probability threshold of 0.5.The bold values are the optimal indicator results of different resolutions.

The performance of the baseline model, the proposed model, and general ophthalmologist to identify whether the posterior pole is involved or not is shown in Table 3. The general ophthalmologist had an 86.49% (95%CI: 85.85–87.10%) sensitivity and an 86.30% (95%CI: 85.63–86.93%) specificity, whereas the model had an 82.49% (95%CI: 81.50–83.44%) sensitivity and a 91.16% (95%CI: 90.34–91.91%) specificity with a probability threshold of 0.5. Despite a high specificity of 91.89% (95%CI: 91.13–92.59%) achieved, the baseline model showed limited sensitivity of 74.53% (95%CI: 73.39–75.64%) for early identification.

**Table 3:** The localization of RD in the posterior pole area by the weakly-supervised deep learning model and the general ophthalmologist compared with the ground truth in the test set.

Index	Sensitivity (95%CI)	Specificity (95%CI)	Accuracy (95%CI)
Baseline model (without AMM)^1^	0.7453 (0.7339–0.7564)	0.9189 (0.9113–0.9259)	0.8295 (0.8224–0.8363)
Weakly-supervised model^1^	0.8249 (0.8150–0.8344)	0.9116 (0.9034–0.9191)	0.8651 (0.8586–0.8713)
General ophthalmologist	0.8649 (0.8585–0.8710)	0.8630 (0.8563–0.8693)	0.8639 (0.8593–0.8683)

^1^The localization performance of the weakly-supervised deep learning model was evaluated with a probability threshold of 0.5. The image resolution of the input data is 512 × 512 pixels.AMM, attention modulation module.

**Figure 3:**
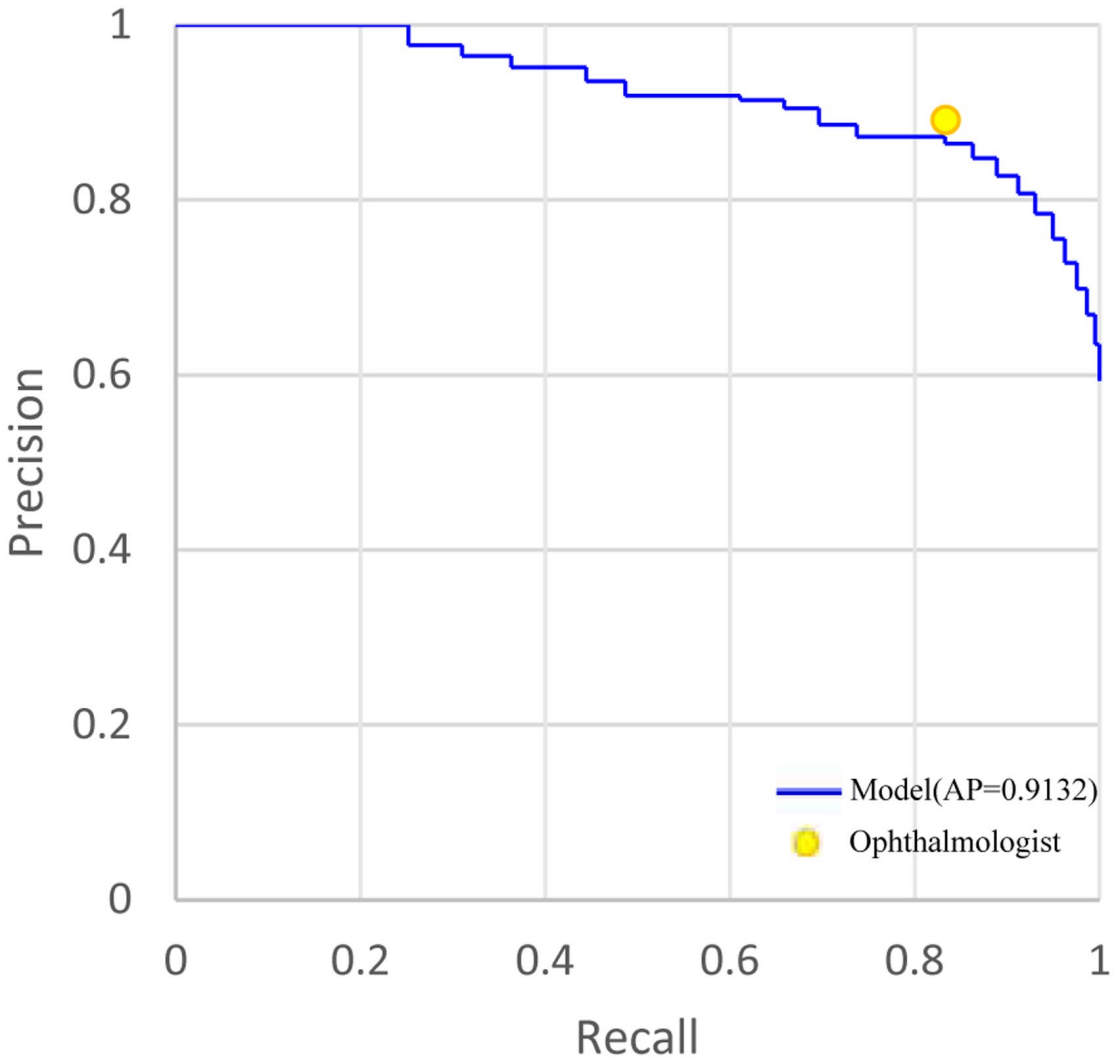
The precision-recall curve of holistic localization performance of RD region based on the weakly-supervised model and general ophthalmologist, compared with the ground truth in the test set. AP, average precision. RD, retinal detachment. AMM, attention modulation module.

As for localizing RD lesions in 48 anatomical regions, the general ophthalmologist had an 89.16% (95%CI: 88.75–89.55%) precision and 83.38% (95%CI: 82.91–83.84%) recall. In contrast, our model had an 86.42% (95%CI: 85.81–87.01%) precision and an 83.27% (95%CI: 82.62–83.90%) recall with an average precision (AP) of 0.9132. Though the baseline model achieved a 92.67% (95%CI: 92.11–93.19%) precision which could be attributed to the most discriminative response region, it showed limited recall of 68.07% (95%CI: 67.25–68.88%). For visualizing the model performance when different probability thresholds are applied, the precision-recall curve of the model is shown in Figure 3. The performance of localizing RD lesions in all 48 anatomical regions by the proposed model and general ophthalmologist is shown in Table 4.

**Table 4:** The performance of localizing RD lesions in 48 anatomical regions by the baseline model, weakly-supervised model, and the general ophthalmologist, compared with the ground truth in the test set.

Index	Precision (95%CI)	Recall (95%CI)	F1 score (95%CI)
Baseline model (without AMM)^1^	0.9267 (0.9211–0.9319)	0.6807 (0.6725–0.6888)	0.7849 (0.7785–0.7912)
Weakly-supervised model^1^	0.8642 (0.8581–0.8701)	0.8327 (0.8262–0.8390)	0.8481 (0.8426–0.8535)
General ophthalmologist	0.8916 (0.8875–0.8955)	0.8338 (0.8291–0.8384)	0.8617 (0.8564–0.8668)

^1^The localization performance of the weakly-supervised deep learning model was evaluated with a probability threshold of 0.5. The image resolution of the input data is 512 × 512 pixels.AMM, attention modulation module.

**Figure 4:**
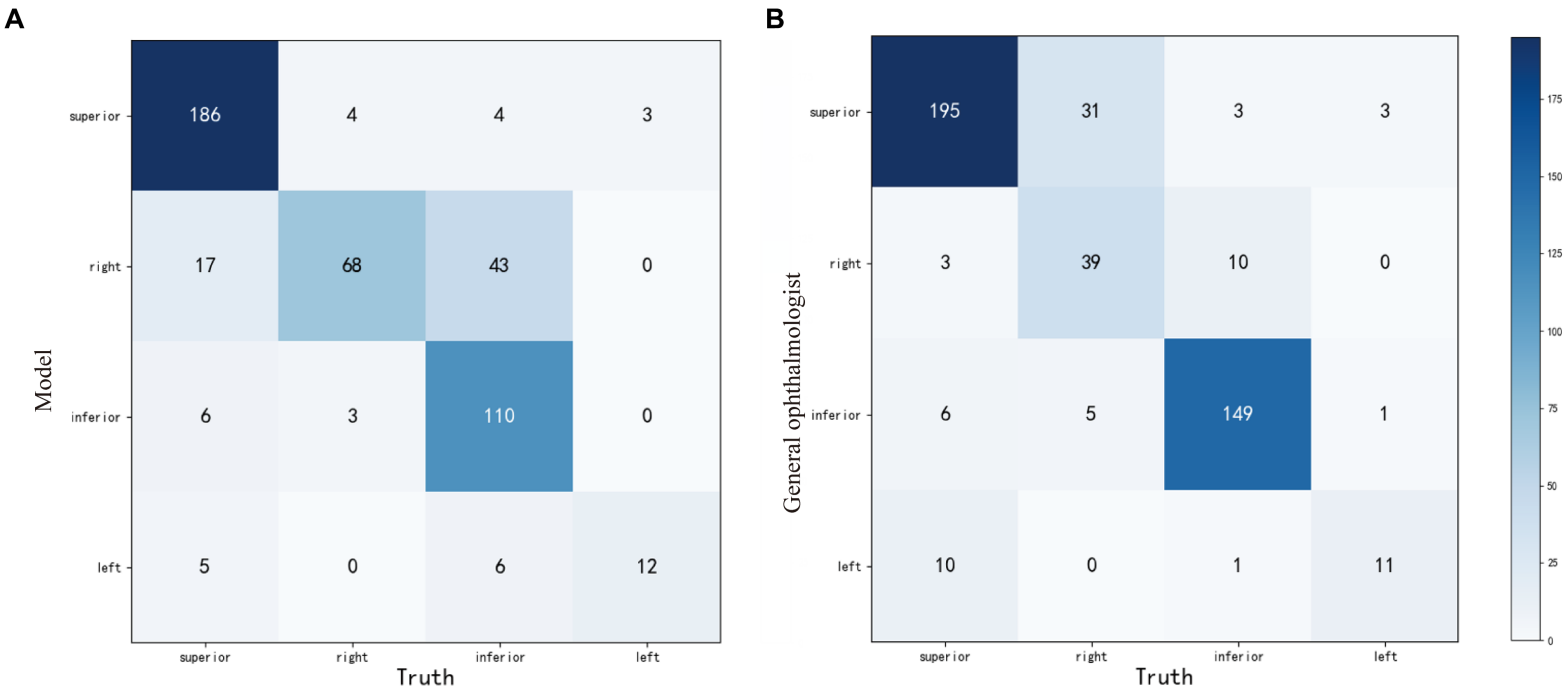
The confusion matrixes of four-zone RD localization performance based on the weakly-supervised model **(A)** and the general ophthalmologist **(B)**, compared with the ground truth in the test set. RD, retinal detachment.

Compared with the ground truth, the unweighted Cohen’s κ coefficients were 0.710 (95%CI: 0.659–0.761) and 0.753 (95%CI: 0.702–0.804) for the weakly-supervised model and the general ophthalmologist, respectively. The four-zone location accuracy of our model is 0.8051 (95%CI: 0.7656–0.8395), which is slightly inferior to the general ophthalmologist’s accuracy of 0.8437 (95%CI: 0.8068–0.8748). The confusion matrixes are shown in Figure 4.

## 4 Discussion

RD is a typical ophthalmic emergency. Early medical interventions based on the precise localization of lesions could increase the success rate of surgical repair and avoid permanent visual impairment (6, 34). Here, we established a standardized procedure for RD localization from UWF images using a weakly-supervised approach. It could provide a corresponding medical reference to both clinicians and RD patients throughout the early-stage management. Compared with the baseline model which only focused on the most discriminative regions with limited recall, our weakly supervised model incorporated the AMR scheme. For this reason, the generated localization maps yielded a comprehensive presentation of RD lesion-related information. The four-zone anatomical localization performance of our model, which was highly related to posture regimens (6, 35, 36), showed substantial consistency with the specialists according to the unweighted Cohen’s kappa coefficients of 0.710(95%CI: 0.659–0.761). The human-model comparisons also demonstrated its localization performance with high precision and recall, almost equaled to a general ophthalmologist’s judging ability. In general, our model exhibits acceptable performance for the holistic localization of the RD regions. To the best of our knowledge, this is the first attempt to precisely localize the RD regions.

Previously, several deep learning systems in identifying RD in fundus images presented favorable performance (16, 17, 37, 38). Similarly, our model also showed a perfect capacity of discernment for RD from UWF images. Nevertheless, previous deep learning models were mainly proposed for classification tasks, and CAMs were employed for post-hoc interpretability. Since such heatmaps were classification-oriented, they tended to resort to some discriminative regions instead of the holistic bound of the whole object. Even though Li et al. (17) attempted to visualize the decisive regions with saliency maps and embedded an arrow according to the hot regions for head positioning guidance, the most decisive regions in the heatmaps may not be the primary location of RD lesions. The classifiers may only focus on a small part of the target lesions (26, 39). Moreover, the limited localization results from true-positive samples had yet to be thoroughly evaluated for general feasibility. In contrast to simply utilizing classification-oriented heatmaps, our model presents the edge of providing lesion-specific holistic activation maps to localize RD regions. For digging out the regions that are essential but easily ignored for lesion segmentation by the weakly supervised algorithm, we introduce AMM to our scheme to provide recalibration supervision and task-specific concepts. The lesion information of clinical interests provided by this interpretable method complies with cognitive law, which could indicate the diagnostic reference to the clinicians and could be verified easily. Moreover, in the coordinates established above, the model could elaborate on the anatomical zones of the RD lesions. According to the most affected zone, a supine preoperative position is advised for RD in the superior zones and a sitting position for RD in the inferior zones (9). Patients with RD lesions in the right or left zones were positioned flat on the right or left side of the affected eye, respectively (40). The involvement of the posterior pole is almost suggestive of a relatively poor vision prognosis if emergency repair surgery is not available before the macula is involved (4, 7, 41). Patients should maintain a supine position during this time and take an urgent referral.

**Figure 5:**
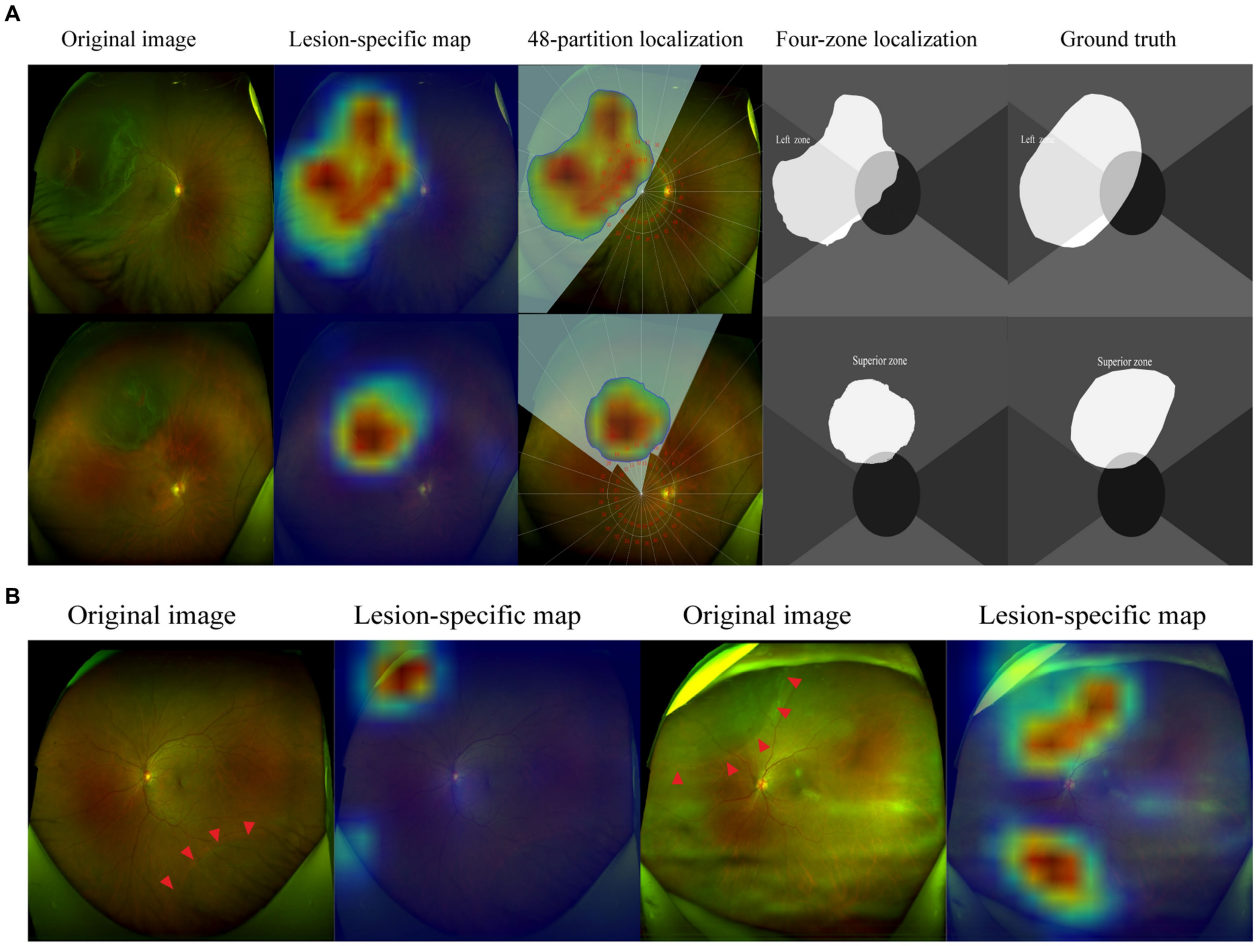
Visualization of representative cases. The corrugated retina and the edge of breaks are highlighted in lesion-specific maps, the detached regions were demonstrated in 48-partition localization maps and four-zone localization maps **(A)**. The shallow retinal detachments are not detected in the inferior quadrant, while OPTOS camera pads are highlighted. Artifacts caused by opaque refractive media are highlighted in localization maps **(B)**. The red arrowheads indicate the borders of the RD region.

In our research, most cases can realize holistic localization of RD lesions with great satisfaction. As shown in Figure 5, the corrugated retinal appearance of RD lesions makes them more distinguishable, whereas the shallow RDs are easily missed due to their atypical appearance. In addition, interference from irrelevant factors can also be misleading for automatic localization. The OPTOS camera pads and artifacts with RD-similar edges may result in mistaken highlights in localization maps. In future work, these problems could be improved by further training based on large-scale images with corresponding issues.

This study has several limitations. First, blurred border and texture feature differences within the RD regions made it difficult for the activation maps to highlight the whole area of the lesion. The regions with inconspicuous texture features were easily missed even though the advanced AMM module had been incorporated, which may result in some inconsistency in anatomical localization. Strictly, the localization of breaks of the rhegmatogenous RD had more significance for posture instructions. However, the small breaks in the retina were not always visible, especially in the peripheral regions. Given most of the breaks are within the detached retina, the localization of the RD region could extend its clinical applications considerably. In addition, the determination of whether the posterior pole was involved may not represent the status of the macula, especially when the macula was located near the borderline of the RD regions. Hence, further work is warranted to accurately discern the status of the macula for determining operation time and predicting visual prognosis. Furthermore, automatic postural guidance had a relatively limited application range due to the high-quality images required for anatomical localization. The anatomical localization of RD was highly dependent on the clear presentation of the retina. Those fundus images with significant opaque refractive media, inappropriate illumination, and invisible optic disc were not eligible for inclusion in this study. Finally, our model was developed based on single-center retrospective datasets with limited generalization. The evaluation of localization accuracy was conducted on a single-disease dataset and was not strictly validated in the cases of fundus comorbidities. In the future, we expect to explore more advanced methods to aid the full-stage management of RD, incorporating the medical history and other imaging data. Meanwhile, we will expand the training samples of fundus comorbidity images and facilitate the evaluation based on the large-scale test scenario.

## 5 Conclusion

In this study, we developed a weakly-supervised deep learning model to localize RD regions based on UWF images. The lesion-specific localization maps could be incorporated into the diagnostic process and personalized postural guidance of RD patients for reference. Moreover, the implementation of this task considerably surmounted the current “label-hunger” difficulty. It would greatly facilitate managing RD patients when insufficient specialists are available, especially for medical referral and postural guidance. The application of this model could significantly equilibrate medical resources and improve healthcare efficiency.

## Data availability statement

The raw data supporting the conclusions of this article will be made available by the authors, without undue reservation.

## Ethics statement

The studies involving humans were approved by Institutional Review Board of the Second Affiliated Hospital of Zhejiang University, School of Medicine. The studies were conducted in accordance with the local legislation and institutional requirements. Written informed consent for participation was not required from the participants or the participants’ legal guardians/next of kin in accordance with the national legislation and institutional requirements. Written informed consent was obtained from the individual(s) for the publication of any potentially identifiable images or data included in this article.

## Author contributions

HL: Conceptualization, Data curation, Formal analysis, Methodology, Software, Writing – original draft. JC: Conceptualization, Data curation, Validation, Writing – review & editing, Writing – original draft. KY: Data curation, Formal analysis, Methodology, Software, Writing – original draft. YZ: Resources, Writing – review & editing. JY: Conceptualization, Funding acquisition, Project administration, Resources, Supervision, Writing – review & editing.
